# Vitamin D deficiency is associated with community-acquired clostridium difficile infection: a case–control study

**DOI:** 10.1186/s12879-014-0661-6

**Published:** 2014-12-04

**Authors:** Tanya Sahay, Ashwin N Ananthakrishnan

**Affiliations:** Division of General Internal Medicine, Massachusetts General Hospital, Boston, MA USA; Division of Gastroenterology, Massachusetts General Hospital, Boston, MA USA; MGH Crohn’s and Colitis center, 165 Cambridge Street, 9th Floor, Boston, 02114 MA USA

**Keywords:** C difficile, Vitamin D, Community-acquired, Infection, Cathelicidin

## Abstract

**Background:**

*Clostridium difficile* infection (CDI) is increasingly recognized as an important community acquired pathogen causing disease (CA-CDI). Vitamin D [25(OH)D] has immune modulatory effects and plays an important role in intestinal immunity. The role of vitamin D in CA-CDI has not been examined previously.

**Methods:**

This was a single referral center case–control study. Cases comprised of all patients with CA-CDI who had a serum 25(OH)D measured within 12 months prior to infection. Controls were drawn from patients who had 25(OH)D checked and matched based on age, gender, race and health status. Serum 25(OH)D was stratified as < 15 ng/mL, 15-30 ng/mL or > 30 ng/mL. Regression models adjusting for potential confounders were used to define independent association between vitamin D and CA-CDI.

**Results:**

We identified 58 matched case–control pairs (66% women; 85% Caucasian). The mean age was 62 years. The mean serum 25(OH)D level was significantly lower in CA-CDI cases compared to controls (28.5 ng/mL vs. 33.8 ng/mL, p = 0.046). Cases had higher rate of antibiotic exposure and more comorbidity. Serum 25(OH)D < 15 ng/mL was associated with an increased risk of CA-CDI on univariate (Odds ratio (OR) 5.10, 95% confidence interval (CI) 1.51 – 17.24) and multivariate analysis (OR 3.84, 95% CI 1.10 – 13.42). Vitamin D levels between 15-30 ng/mL did not modify disease risk.

**Conclusions:**

Low serum 25(OH)D < 15 ng/mL was associated with increased risk of CA-CDI. This suggests vitamin D may have a role in determining susceptibility to CA-CDI.

## Background

*Clostridium difficile* (*C. difficile*) is a gram-positive, anaerobic, spore-forming bacillus that has emerged as a serious and common healthcare-associated infection with significant morbidity [1]-[7]. The spectrum of illness caused by *C. difficile* ranges in severity from asymptomatic or self-limited mild diarrhea, to fulminant colitis and death. *C. difficile* infections (CDI) are costly; a retrospective analysis of Massachusetts hospital discharge data demonstrated a total cost of 55,380 inpatient-days and $51.2 million over 2 years [8]. Based on national estimates of the numbers of patients affected by CDI, the annual cost is roughly $3.2 billion dollars [9]. In addition to the present disease burden, an analysis performed by the Centers for Disease Control and Prevention projects that, in the United States, the number of cases of CDI continues to rise [9].

One key mechanism underlying pathogenesis of CDI is disruption of the host microbial flora, commonly through broad-spectrum antibiotic use [1]-[7]. In observational studies, between 50-95% of patients with CDI had recent exposure to antibiotics or other healthcare environments facilitating transmission of *C difficile* [1]. Other risk factors remain less well established including use of acid suppressive medications [10],[11], underlying inflammatory bowel disease [12],[13], pregnancy or post-partum state [14]-[17], and liver disease [18]-[20]. Yet much regarding the host risk factors for CDI remains inadequately defined. Host immune response to *C difficile* in the form of antibody production is associated with reduced rates of carriage [21]. Host genetic factors governing immune response, particularly innate immunity, may also play a role in determining susceptibility to CDI [22]. Consequently, factors that influence such host immune responses may additionally contribute towards the pathogenesis of CDI.

A recent factor associated with CDI in the hospitalized population and in those with inflammatory bowel disease (IBD) is deficient plasma vitamin D [23]-[25]. There is increasing interest in the immunological role of vitamin D particularly on the innate immune response [26]-[28]. Cathelicidins are antimicrobial peptides whose production is stimulated by vitamin D [29]-[31]. In laboratory studies, administration of exogenous cathelicidin reduced severity of *C difficile* infection [30]. Limited data supports such an association in humans. One study demonstrated an association between low plasma vitamin D and CDI associated health-care costs [32], while more recently Quraishi *et al*. reported an association between reduced pre-hospital plasma 25-hydroxy vitamin D and increased risk for hospital acquired CDI (HA-CDI) [23]. There is increasing recognition that a substantial portion of CDI may occur in the community (CA-CDI) in individuals without the well-recognized risk factors for CDI [33],[34]. The role of vitamin D deficiency in predisposing to disease risk in this population has not been examined previously but yet is an important question to examine as risk factors in the hospitalized patients who tend to be older and have greater co-morbid burden may not be applicable to individuals in the community. In this retrospective case–control study, we examined the association between reduced plasma 25-hydroxy vitamin D and risk of CA-CDI.

## Methods

### Data source and patient population

The primary source of data for this study was electronic medical record (EMR) data generated during patient care episodes at Massachusetts General Hospital, a tertiary referral center in the greater Boston metropolitan area. The cases were identified by querying an internally-maintained database of EMR data (Research Patient Data Repository, RPDR) for patients seeking care between Jan 1, 2010 and June 30, 2013 who had a positive laboratory assay for *C. difficile* toxin antigen in the stool. A diagnosis of CDI was established by symptoms in combination with detection of *C. difficile* toxin in the stool using enzyme linked immunosorbent assay (ELISA) except during the last year of the study when the testing algorithm switched to a two-stage strategy with initial screen using the ELISA for glutamate dehydrogenase (GDH) followed by the toxin assay with the use of polymerase chain reaction (PCR) for final determination in the setting of indeterminate toxin assay. The RPDR is an electronic database that is automatically and continuously populated with every patient encounter, laboratory test, radiologic or operative procedure at a Partners Healthcare affiliated facility, and incorporates data from billing codes, clinical laboratory, inpatient, and outpatient stays [35]. For the purpose of this study, a case of CA-CDI was defined as an outpatient with a positive assay or inpatients who had a positive test within the first 48 hours of hospital admission, consistent with the definition of CA-CDI [36]. Patients with prior hospitalization or stay at a healthcare-associated facility within the past 90 days were excluded [36]. Cases were included if they had 25-hydroxy vitamin D [25(OH)D] measured within 1 year before or within 2 weeks after toxin positivity. We allowed for a 2 week window for assessment of vitamin D status after diagnosis as levels of 25(OH)D are unlikely to change immediately after an acute infection, and levels measured within this window are likely reflective of pre-diagnosis vitamin D [37],[38]. Controls were matched to cases in a 1:1 ratio by age, sex, race, and health status (defined via the number of health care visits in a given period of time). None of the included patients had a prior history of CDI.

### Variables

The exposure of interest was vitamin D status assessed as the 25(OH)D level closest in proximity to the date of toxin positivity. As noted above, levels from one year prior to admission to two weeks after toxin positivity were allowed. In order to define cutoffs, we noted that in the previous study performed by Quraishi *et al*., there was an increased risk of HA-CDI only with 25(OH)D levels <10 ng/mL, however there were very few cases that had 25-OH-Vitamin D levels in this range [23]. As a result, we decided to combine the bottom two groups and use the midpoint to arrive at a cutoff of 15 ng/mL with values below this level representing vitamin D deficiency. Additionally, we used 30 ng/mL and above as normal per Institute of Medicine guidelines. Individuals with 25(OH)D levels between 15-30 ng/mL represent vitamin D insufficiency. Plasma 25(OH)D was measured using high-performance liquid chromatography with mass spectrometry. This has previously demonstrated a low co-efficient of variance and has been used in prior studies from our institution [39].

Information was collected including age, gender, race and comorbidity. Comorbidity was assessed using the widely-used and validated Charlson Comorbidity Index (CCI), which was computed using the recorded billing codes for the patient prior to their CA-CDI encounter [40]. Medication use was assessed using the electronic prescription function of the EMR. Specifically prescriptions for antibiotics, proton-pump inhibitors (PPI), vitamin D supplements, multivitamins, and statins within the 90 days prior to admission were noted. These variables were selected because multivitamin represent a source of vitamin D supplementation and could be a confounder for ‘healthy behaviors’ while statins have been previously shown to be inversely associated with risk of CDI [41]. Other laboratory values collected included the white blood cell (WBC) count on admission, peak WBC count, baseline serum creatinine level or need for dialysis, peak serum creatinine level, and serum albumin.

### Statistical analysis

Statistical analyses were performed using the JMP Platform (JMP 11 Professional, SAS Institute, Cary, NC) and Stata SE 12.1 (StataCorp, College Station, TX). Continuous variables were summarized using means and standard deviations, and compared using paired t-tests. Categorical variables were expressed as proportions and compared using Fisher’s exact tests. Plasma 25(OH)D levels were modeled as a categorical variable with three levels (as described above, normal, insufficiency, and deficiency). Multivariate logistic regression was performed to adjust for potential confounders, the final model included variables significant at p < 0.10 on univariate analysis. Logistic regression models were created with and without adjustment for antibiotic use as vitamin D deficiency has been associated with increased risk of infections and consequently antibiotic use could represent an intermediate step in the pathway of development of CDI rather than a true confounder [23],[26]. Ethical approval was obtained from the Institutional Review Board of Partners Healthcare.

## Results

Fifty-eight cases of CA-CDI were successfully matched with control patients. The demographic and clinical characteristics of these groups are presented in Table 1. Two-thirds were women (66%), and a majority was Caucasian (85%), with a mean age of 62 years (standard deviation (SD) 19 years). The mean 25(OH)D levels were 28.5 ng/mL (SD 15.4) in the cases, compared to 33.8 ng/mL (SD 12.8) in the controls (p = 0.046). The cases demonstrated higher rates of prior exposure to antibiotics, statins, and proton pump inhibitors (PPI), and greater comorbidity.

**Table 1:** **Characteristics of patients with community acquired Clostridium difficile infection (CA-CDI) and matched controls**

		Cases (n = 58)		Controls (n = 58)		p-value
**Age (in years)**	Mean (SD)	62.1	(19.2)	62.2	(19.7)	0.99
**Female**	n (%)	38	(65.5)	38	(65.5)	1.00
**Caucasian Race**	n (%)	49	(84.5)	49	(84.5)	1.00
**Charlson Index**	Mean (SD)	6.19	(4.16)	1.33	(1.63)	< 0.01
**Antibiotic Use**	n (%)	45	(77.6)	14	(24.1)	< 0.01
**Statin Use**	n (%)	18	(31.0)	7	(12.1)	0.013
**PPI Use**	n (%)	33	(56.9)	6	(10.3)	< 0.01
**Supplement vitamin D use**	n (%)	22	(37.9)	10	(17.2)	0.013
**Multivitamin Use**	n (%)	17	(29.3)	3	(5.2)	0.001
**Baseline Albumin (g/dL)**	Mean (SD)	3.21	(0.77)	4.30	(0.57)	< 0.01
**Baseline Creatinine (mg/dL)**	Mean (SD)	2.37	(6.74)	0.95	(0.37)	0.12
**Plasma 25(OH) D (ng/mL)**	Mean (SD)	28.5	(15.4)	33.8	(12.8)	0.04

SD – standard deviation; PPI – proton pump inhibitor.

Univariate analysis of the variable of interest and the primary potential confounders is presented in Table 2. Vitamin D deficiency was strongly associated with CA-CDI, with an OR of 5.1(95% CI 1.51 – 17.24) for those with levels below 15 ng/ml when compared to those with levels > 30 ng/mL. Vitamin D insufficiency, i.e. levels between 15-30 ng/mL was not associated with CA-CDI, with OR of 1.22 (95% CI 0.54 – 2.78, p = 1.0). Other variables associated with CA-CDI were use of antibiotics (OR 10.88, 95% CI 4.60 – 25.75), statins (OR 3.28, 95% CI 1.25 – 8.62), and PPI (OR 11.44, 95% CI 4.24 – 30.85).

**Table 2:** **Univariate logistic regression of predictors of community acquired** ***clostridium difficile*** **infection**

	Odds ratio (OR)	95% confidence interval	p-value
**Plasma 25(OH)D**			
> 30 ng/mL	1.0		
15-30 ng/mL	1.22	0.54 – 2.78	0.63
<15 ng/mL	5.10	1.51 – 17.23	0.009
**Charlson Index ≥1**	4.92	1.81 – 13.38	0.002
**Antibiotic Use**	10.88	4.60 – 25.75	< 0.001
**Statin Use**	3.28	1.25 – 8.62	0.016
**Proton pump inhibitor use**	11.44	4.24 – 30.85	< 0.001
**Baseline Albumin (for each 1 g/dL increase)**	0.11	0.04 – 0.24	< 0.001
**Baseline Creatinine (for each 1 mg/dL increase)**	1.85	1.07 – 3.16	0.027

The final logistic regression model adjusting for potential confounders is presented in Table 3. After adjustment for season of measurement and comorbidity (with matching on age and gender), vitamin D deficiency continued to be independently associated with CA-CDI (OR 3.84, 95% CI 1.10 – 13.43, p = 0.035). The addition of antibiotic exposure within the previous 90 days only weakly attenuated the association between CA-CDI and vitamin D deficiency (OR 3.77, 95% CI 0.92 – 15.47, p = 0.066). Vitamin D insufficiency was not associated with CA-CDI in either model.

**Table 3:** **Multivariate logistic regression of predictors of community acquired** ***clostridium difficile*** **infection**

	Odds ratio (OR)	95% confidence interval	p-value
**Plasma 25(OH)D**			
> 30 ng/mL	1.0		
15-30 ng/mL	1.09	0.45 – 2.62	0.845
<15 ng/mL	3.84	1.10 – 13.42	0.035
**Charlson Index ≥1**	4.13	1.47 – 11.61	0.007
**Winter**	1.00	0.44 – 2.28	0.995

+Model adjusting for co-morbidity, season of vitamin D measurement. Patients were additionally matched on age, gender, and health status.

## Discussion

Recent studies have highlighted the immunological role of vitamin D, its role in intestinal inflammation including Crohn’s disease, and in determining susceptibility to infections [26],[28]. Furthermore, intriguing emerging data has supporting a potential role for vitamin D deficiency in influencing susceptibility to CDI [23],[25] and amelioration of severity of CDI by administration of exogenous cathelicidin, an antimicrobial peptide induced by vitamin D [30]. In our study, we demonstrate that 25(OH) D levels of <15 ng/mL are associated with an increased risk for CA-CDI. This effect persists even after adjusting for several potential confounders, supporting our hypotheses that vitamin D may play a role in gut immunity, and that vitamin D deficiency may predispose patients to CA-CDI.

Only few studies have examined an association between vitamin D levels and susceptibility to CDI; they have focused on the role of vitamin D deficiency in mediating susceptibility to CDI in hospitalized patients [23], and in those with IBD [25]. However these findings cannot be generalized to our cohort for several reasons. Compared to those who acquire CDI in the hospital, those with CA-CDI have less comorbidity and fewer risk factors such as antibiotic exposure predisposing to disruption of the enteric microbiome. Similarly, the patient population with IBD-CDI arguably has altered gut immune responses and microbiome when compared to those in the community who develop CDI. Two studies have examined the effect of vitamin D deficiency on CDI outcomes. From a hospitalized cohort with *C difficile* infection, van der Wilden *et al*. noted a higher frequency of vitamin D deficiency in those with severe disease defined as abdominal computed tomography scan findings of colitis [24]. A prior study examining healthcare costs suggested that vitamin D-deficient patients with CDI incurred costs more than five times higher than the non-deficient patients, had longer hospital stay and greater number of hospitalizations [32]. In our study, we did not identify any association between vitamin D deficiency and severity of CA-CDI, but larger cohorts are likely required to more accurately define this association.

Considerable evidence supports this hypothesis at the molecular level. Most cells involved in immune responses including B-lymphocytes, T-lymphocytes, dendritic cells, and monocytes carry vitamin D receptors (VDR) [26],[28]. Furthermore, vitamin D stimulates the expression of potent antimicrobial peptides, cathelicidins. Cathelicidins are a family of peptides thought to be part of an innate defensive barrier against potential microbial pathogens (bacteria, viruses, fungi alike) and are present at skin and mucosa of the gastrointestinal and respiratory tracts [30],[31],[42],[43].

In a recent study, Hing *et al*. demonstrated reductions in *c difficile* related colonic damage at the histologic level, cell apoptosis, tissue myeloperoxidase levels and tumor necrosis factor α levels with cathelicidin administration in wild-type mice [30]. Also using mouse models, Ooi *et al*. showed that vitamin D availability combined with normally functioning vitamin D receptors is required for the development of regulatory T-lymphocytes [44]. With vitamin D- or vitamin D-receptor deficiency, these immune responses are impaired and affected mice are susceptible to immune-mediated diseases of the intestinal tract [44]. Additionally, vitamin D protects macrophages against death from *C difficile* toxin- induced intestinal injury [45]. All of these mechanisms in addition to others yet undiscovered may help explain the association between vitamin D deficiency and CDI.

Further implications of our findings include the potential impact preventive medicine can play curtailing the rates of CDI. Taken together, both HA-CDI and CA-CDI appear to be associated with vitamin D deficient status. If additionally confirmed in other cohorts, routine screening for vitamin D deficiency in primary care offices (already under way in many practices in the United States) can lead to vitamin D supplementation where indicated and achieve a vitamin D replete state. In light of the substantial costs associated with CDI, this outpatient intervention could potentially have significant financial implications, particularly in groups at high risk for CDI. Thus, whether there is a role for routine supplementation of vitamin D, either in primary prevention, or in improvement of outcomes in those with established disease merit further study. As well, mechanistic studies examining the downstream effects of vitamin D, either through the vitamin D receptor or through stimulation of production of cathelicidins, may help shed further light on host immune factors that influence susceptibility to *C difficile* infections.

The most significant limitation of this study is the small sample size. Although this did not impact the observed effect size, it did limit the ability to adjust for multiple confounders. Additional limitations include the retrospective nature of the analysis, which introduces some degree of bias due to completeness of documentation at the time of the original clinical encounter. Also, measurement of vitamin D levels was as part of routine clinical care. Thus, the population having a measured level may differ from the general population in community. However, we would expect this to likely be similar across both the case and control population and not result in a biased association. As in any observational study, there is the possibility for unmeasured confounders. We were able to adjust for most of the relevant variables including age, co-morbidity, antibiotic use and recent healthcare exposure. Additionally, this is a cross-sectional study, which does not allow for estimation of absolute risks or inference of causality. Finally, it is possible that patients who initially had deficient vitamin D levels were recommended supplementation which may have increased their levels by the time of development of CA-CDI. However, we believe this is unlikely to influence our findings for two reasons. Our results were robust on adjustment for supplemental vitamin D or multivitamin use, two commonly recommended methods for supplementing vitamin D levels. Second, such misclassification would actually bias our results towards the null making our effect sizes a more conservative estimate.

## Conclusions

In summary, patients with CA-CDI were more likely to have vitamin D deficiency (<15 ng/mL), and this association persisted as an independent risk factor in logistic regression models even after adjusting for potential confounders. Further prospective studies are needed to confirm our findings. As well, additional studies are also needed to establish mechanisms through which vitamin D mediates gut immunity and susceptibility to enteric infections and intestinal inflammation. Human studies of the role of vitamin D assessment in supplementation, particularly in those at high risk of *C difficile* infection, may be warranted supported by the emerging literature in this field.

### Clinical relevancy statement

*Clostridium difficile* infection (CDI) is an important cause of morbidity and mortality. While commonly recognized as a hospital acquired infection, up to 40% of CDI could be acquired in the community by individuals with no extrinsic risk factors. Thus, identification of potential host factors that predispose to CDI is an important goal. In this study, we demonstrate low vitamin D to be associated with increased risk of CDI. Once confirmed in additional independent cohorts, this may form a basis for future clinical trials of vitamin D in the prevention and treatment of CDI. It may also help laboratory studies aiming to understand the host response to *C difficile*. This finding is clinical relevant to guide clinicians to routinely monitor vitamin D status in those at risk for *C difficile*.

## Financial conflicts of interests

Dr. Ananthakrishnan has served on the scientific advisory boards for Cubist pharmaceuticals and Abbvie.
